# Effect of psyllium husk (*Plantago ovata*) on the physicochemical and textural properties of chicken model sausages and its application in an *in vitro* digestion model

**DOI:** 10.5713/ab.260344

**Published:** 2026-07-27

**Authors:** Ji Seon Choi, Koo Bok Chin

**Affiliations:** 1 Department of Animal Science, Chonnam National University, Gwangju, Korea

**Keywords:** Chicken, *In Vitro* Digestion, Physicochemical Properties, Psyllium Husk, Sausage

## Abstract

**Objective:**

This study aimed to evaluate the quality characteristics of chicken model sausages based on the amount of added psyllium husk powder (PHP; 0%, 0.5%, 1.0%, and 1.5%) and to determine protein digestibility using an *in vitro* digestion model.

**Methods:**

The effect of PHP on the quality of chicken model sausages was assessed in terms of physicochemical properties and protein digestibility.

**Results:**

As the PHP levels increased, lightness decreased and yellowness increased in a concentration-dependent manner (p<0.05). An increase in PHP concentration was also associated with a reduction in cooking loss (CL) and expressible moisture (EM) (p<0.05), demonstrating that PHP possesses water-holding capacity (WHC). No textural property differences were observed among the treatments (p>0.05). Furthermore, sausages containing 1.0%–1.5% PHP exhibited improved protein digestibility during *in vitro* digestion (p<0.05). These findings indicated that PHP improved certain quality attributes in sausages, including WHC and digestibility, without changing texture. PHP addition showed negative correlations with CL, lightness, EM, and springiness, and positive correlations with yellowness and protein digestibility.

**Conclusion:**

This study suggests that PHP can be used as a novel functional ingredient in meat products and may positively impact *in vitro* protein digestibility.

## INTRODUCTION

Psyllium’s seed husk, with its excellent water-absorbing and gelling properties, is a potential novel functional ingredient in food production [1]. Psyllium (*Plantago ovata* Forsk.) is also known as ispaghula or isabgol. The main monosaccharide constituents of psyllium husk mucilage are xylose and arabinose, and it comprises complex side chains composed of minute amounts of galacturonic acid, galactose, and/or rhamnose, among others [2,3]. It was reported that psyllium husk polysaccharides consist primarily of arabinoxylans with complex side-chain structures and are therefore classified as heteroxylans [1]. Previous studies were reported that its gelling ability was related to feruloylated arabinoxylans obtained through the covalent cross-linking of ferulic acid residues in water-extracted arabinoxylans from wheat [4,5]. Therefore, the moisture absorption and gelling properties of psyllium husk make it applicable as a functional material in meat products to potentially enhance final yield and positively affect textural properties. Moreover, psyllium effectively improves gastrointestinal disorders (e.g., constipation, irritable bowel syndrome, inflammatory bowel disease, etc.) and metabolic conditions (e.g., obesity, diabetes, hypercholesterolemia, etc.) and provides cancer prevention, satiety, and prebiotic benefits [6]. Therefore, the application of psyllium husk in meat products may be utilized as a functional additive to enhance quality characteristics, including water-holding capacity (WHC) and health-promoting properties.

Despite the growing body of literature on the application of psyllium husk to myofibrillar protein (MP) gels, which are crucial components of meat proteins, studies exploring its use in meat products remain limited. Previous studies were reported that the addition of psyllium husk promoted interactions between proteins to form a dense MP gel network, thereby improving textural properties and WHC [7,8]. Adding psyllium husk to lamb MP gels improved their textural properties and WHC, even at low NaCl levels [9]. In addition, the addition of psyllium husk potentially improved not only the gel strength and WHC, but also the emulsion stability of MP-based emulsions [10]. Therefore, based on previous studies, applying psyllium husk to meat products is expected to positively affect their texture, WHC, and emulsification. Literature on the exclusive application of psyllium husk to meat products is limited. The addition of psyllium husk (4%, 6%, and 8%) to chicken rolls was reported to increase cooking yield as psyllium husk levels increased [11]. However, texture analysis and protein digestibility were not analyzed. Since previous studies focused on high concentration levels (4%–8%), the effects at lower concentrations remain to be investigated. In addition, previous studies have investigated the effects of psyllium husk addition in meat products (emulsified meat batter or luncheon meat) under reduced-phosphate or none-phosphate conditions. However, to our knowledge, no studies have examined the effects of psyllium husk addition alone or its application in low-fat meat products [12,13]. Due to the lack of research data, studies investigating the development of low-fat meat products added with psyllium husk and evaluating their physicochemical properties are needed.

Assessing the digestibility or bioavailability of nutrients is equally essential, as they influence the metabolism of absorbed nutrients [14]. To evaluate these parameters, the *in vitro* digestive system serves as a critical tool [15]. However, since research on the effects of psyllium husk addition on protein digestibility of meat products is currently lacking, this study is expected to provide valuable insights into the impact of psyllium husk on not only the quality characteristics but also the protein digestibility of meat products. Therefore, this study aimed to evaluate the physicochemical properties of low-fat chicken model sausages containing different concentrations of psyllium husk powder (PHP; 0%, 0.5%, 1.0%, and 1.5%) and investigate the impact of PHP on protein digestibility using a human *in vitro* digestion model. This study further demonstrated that psyllium husk can be effectively applied as a functional ingredient in meat products.

## MATERIALS AND METHODS

### Materials

Raw chicken breasts were purchased locally and stored at −70°C. They were thawed overnight at 4°C and subsequently ground using a meat grinder (M-12S; Hankook Fujee Machinery) to prepare chicken model sausages. Psyllium husk, imported from India, was purchased from Naemomeherbs. To produce PHP, the psyllium husk was ground and passed through a 300-μm mesh sieve (Daehan Science).

### Manufacture of low-fat chicken model sausages

Chicken model sausages were prepared according to a previously described method [16], and the formulation is detailed in Table 1. Briefly, ground chicken meat was mixed with ice water and other ingredients using a mixer (HMC-401; Hanil Electric) for 3.5 min. Thereafter, the batter (40 g) was placed in 50-mL conical tubes (SPL Life Science) and centrifuged at 1,660×g for 2 min (VS-5500; Vision Science). The sausage batter was subsequently cooked at 75°C for 30 min in a water bath (WB-22; Daihan Scientific) and immediately cooled on ice. And then, the cooked sausages were stored at 4°C until further use.

### pH and color values

The pH values of the chicken sausages were measured five times using a pH meter (MP-120; Mettler-Toledo) and the average was calculated. Color values (CIE L* [lightness], CIE a* [redness], and CIE b* [yellowness]) were measured six times from the inner surface of the sausages using a colorimeter (CR-10; Minolta).

### Proximate composition

The proximate composition (%) of the sausages was determined using AOAC methods [17]. For proximate composition analysis, the sausages were ground. Moisture, crude fat, and crude protein content (%) were measured using the dry oven, the Soxhlet extraction, and Kjeldahl methods, respectively.

### Cooking loss and expressible moisture

Cooking loss (CL, %) was calculated based on the weight difference before and after cooking using the following equation:


(1) $$\begin{array}{l} \text{CL }(\%)=(\text{Sample weight }[\text{g}]\;\text{before cooking}-\\ \text{Sample weight }[\text{g}]\;\text{after cooking})/\\ (\text{Sample weight }[\text{g}]\;\text{before cooking})\times 100 \end{array}$$

Expressible moisture (EM, %) was measured based on the method described previously [18]. Briefly, cube-shaped sausage samples (1.5 g) were wrapped in three pieces of filter paper (Whatman #3), centrifuged at 1,660×g for 15 min, and then subjected to weight measurement. EM was calculated using the following equation:


(2) $$\begin{array}{l} \text{EM }(\%)=(\text{Filter paper weight after centrifugation}-\\ \text{Initial filter paper weight})/\text{Initial sample weight}\times 100 \end{array}$$

### Texture profile analysis

Pillar-shaped samples (height: 13 mm, diameter: 12.5 mm) were prepared using a puncturing tool (diameter: 12.5 mm). Texture profile analysis of chicken sausage was performed using an Instron Universal Testing Machine (Model 3344 testing device). Sample height was reduced to 75% of the original height after two sequential compressions. A compression probe with a 500-N load cell was used, and the crosshead speed was set at 300 mm/min. Hardness (gf), springiness (mm), gumminess, chewiness, and cohesiveness were measured.

### Application of *in vitro* digestion model and evaluation of digestibility

An *in vitro* human digestion model was employed following a previously described method [19], with slight modifications. Ground sausage samples (exactly 5 g each) were weighed into 50-mL conical tubes. The sausage samples were shaken for 5 min in a shaking water bath (150 rpm, 37°C) containing 5 mL of saliva (α-amylase [290 mg/L] and mucin [25 mg/L], pH 6.8) for oral digestion. For gastric digestion, 10 mL of gastric juice (pepsin [2.5 g/L] and mucin [3 g/L], pH 1.5) was added to the oral digestion sample and shaken for 2 h in a shaking water bath under similar conditions. For small intestinal digestion, the samples were each reacted with 10 mL of duodenal juice (pancreatin [9 g/L] and lipase [1.5 g/L], pH 8.0) and 5 mL of bile juice (bile [30 g/L], pH 7.0) for 2 h. After digestion, they were immediately centrifuged at 12,000×g for 5 min at 4°C. The supernatant was immediately used to calculate protein digestibility (%) according to the method described previously [20], with slight modifications. Protein digestibility (%) was calculated based on the difference in protein concentration between the sausage before digestion (*A*) and the digesta supernatant (*B*) using the following formula:


(3) Protein digestibility (%)=B/A×100

### Sodium dodecyl sulfate–polyacrylamide gel electrophoresis

Sodium dodecyl sulfate–polyacrylamide gel electrophoresis (SDS-PAGE) was performed using 10% or 12% polyacrylamide separating gels and 4% stacking gel prepared using Mini-PROTEAN 3 Cell (Bio-Rad). Both the initial and digested residues of chicken sausages were dissolved in a 5% SDS solution. The supernatant’s protein concentration was measured using a Pierce BCA protein assay kit (#23227; Thermo Fisher Scientific). Each loading sample (15 μL) contained 15 μg of protein and 2× Laemmli sample buffer (#161-0737; Bio-Rad). Precision Plus Protein Standards (#161-0373; Bio-Rad) were used as protein markers.

### Low‐vacuum scanning electron microscopy

Chicken sausages were fixed and sequentially dehydrated using 2.5% glutaraldehyde (in 0.1 M sodium phosphate buffer, pH 7), 1% osmium tetroxide (in 0.1 M sodium phosphate buffer, pH 7), various levels of ethanol (50%, 60%, 70%, 80%, 90%, and 100%), and acetone. The dried sausage samples were coated with gold using an auto sputter coater (Model 108; Cressington Scientific Instruments). The three-dimensional structure of the digested sausages was observed utilizing a low-vacuum scanning electron microscopy (LV-SEM, JSM-6610LV; JEOL).

### Statistical analysis

Experimental data (one-way analysis of variance, ANOVA) and Spearman’s correlation coefficients were analyzed using IBM SPSS Statistics 27.0 software (IBM). The sausage treatments containing different levels of PHP were each independently manufactured three times (n = 3), and all physicochemical properties and *in vitro* protein digestibility were measured from each independently manufactured batch. Significant differences in the one-way ANOVA were determined using Duncan’s multiple range test at a significance level of p<0.05. A correlation heatmap was generated in Excel 2019 (Microsoft).

## RESULTS AND DISCUSSION

### pH and color values

The pH and color values of chicken sausages containing various levels of PHP are shown in Table 2. The pH values ranged from 6.26 to 6.29, exhibiting no significant difference according to PHP concentration (p>0.05). This indicates that PHP addition did not affect sausage pH value. A previous study also found that adding psyllium husk to chicken meat rolls did not affect the pH value of either the meat batter or the final product [11]. Previous studies have reported that the incorporation of certain dietary fibers (e.g., cellulose nanofibers and wheat fiber) into meat systems has no effect on pH [21,22]. Conversely, in another study, the addition of bamboo shoot dietary fiber decreased the pH of pork meat batter, possibly due to the acidic compounds it contains [23]. Therefore, the effect on the pH of meat products may vary depending on the characteristics of the added dietary fiber. In our study, the addition of up to 1.5% PHP to meat systems did not alter the pH level.

In terms of sausage color, the lightness (CIE L*) and yellowness (CIE b*) values of the sausage decreased in a PHP concentration-dependent manner (p<0.05). No differences in redness (CIE a*) were found among all treatments *(*p>0.05). In a previous study, the dietary fiber (inulin, oat, and psyllium) in chicken sausages decreased in lightness, whereas psyllium husk increased in yellowness and redness [24]. However, in our study, redness did not differ, possibly because the previous study used a higher psyllium husk content (3% or 6%). These changes may result from the inherent color of PHP, and the degree of its impact on the color of meat products potentially varies with the quantity added. In another study, as the concentration of water-dispersed psyllium husk increased, both in sol and gel states, the lightness, redness, and yellowness values also tended to increase [25]. A previous study reported that adding bamboo shoot dietary fiber to pork products resulted in a decrease in L* and an increase in b* [23]. The addition of oven-dried lotus root powder decreased the L* value of regular-fat model sausages, while increasing the a* and b* values due to its inherent color (reddish-yellow) [26]. These changes may be attributed to either the color and content of the added dietary fiber or the dietary fiber-induced reduction in the CL of the meat product.

### Proximate composition

The proximate compositions of chicken sausages supplemented with various concentrations of PHP are listed in Table 3. No differences in moisture, crude fat, or crude protein content were noted (p>0.05). A previous study demonstrated that increasing the psyllium husk concentration reduced the moisture and protein content of chicken meat rolls, possibly because of the replacement of lean meat with psyllium husk [11]. However, in our study, no difference in moisture and protein content was observed, possibly because the maximum PHP concentration in our study was 1.5%, which was lower than the 4%–8% used in previous studies [11]. In another study, replacing pork back fat with cellulose nanofibers did not affect the protein content of cooked meat batter because the same amount of lean meat was added [21]. Similarly, in our study, the amounts of ingredients used during sausage manufacture were the same, except for PHP. Therefore, the moisture, fat, and protein contents were presumably unaffected by PHP addition of up to 1.5%.

### Cooking loss and expressible moisture

The CL and EM results of chicken sausages supplemented with varying amounts of PHP are shown in Figure 1. As the PHP level increased up to 1.5%, CL from the sausages decreased (p<0.05). This can be attributed to PHP’s excellent moisture absorption ability, which effectively prevents moisture loss during cooking and improves the final yield of the meat product. Psyllium husk is primarily composed of arabinoxylan, a comb-like polysaccharide with xylose and arabinose residues densely arranged as side chains, resulting in a highly branched structure. Arabinoxylan, the main component of psyllium husk, forms an entangled structure through hydrogen bonding with water molecules during gelation, owing to its branched side chains and abundant hydroxyl (–OH) groups [3]. This interaction could lead to swelling and the formation of mucilage and gel-like structures. Increasing the quantity of psyllium husk added to chicken meatballs has also been reported to effectively reduce CL, thereby increasing product yield [11]. In another study, the addition of bamboo shoot dietary fiber reduced CL and expressible water loss from pork meat products owing to the water-binding capacity of dietary fiber or a decrease in the amount of water added as the quantity of dietary fiber increased during meat product manufacture [23]. In this study, since the amount of water added to the meat batter was consistent, it appears that CL was effectively reduced by the water-binding capacity of PHP.

As shown in Figure 1B, the addition of PHP (0.5%–1.5%) led to a reduction in EM compared to sausages without PHP (p<0.05). Notably, the lowest EM was observed in samples containing 1.0–1.5% PHP (p<0.05). PHP addition not only effectively prevented CL owing to its water-binding ability, but also reduced EM (p<0.05). This suggested that PHP is potentially useful as a binder that can increase product yield by improving WHC during processing. A previous study reported that the WHC of psyllium husk allowed it to absorb 45.03 g of water per gram of dried sample, which is approximately 4–5 times higher than that absorbed by sugarcane fiber [27]. This supports our results wherein CL and EM were reduced in PHP-added sausages owing to PHP’s remarkable WHC. In a previous study [25], psyllium husk yielded WHC and water-retention capacity values of 27 and 25.8 g water/g dry sample, respectively, suggesting that the amount of water absorbed by psyllium husk and retained under centrifugal force was closely comparable. PHP can bind to water molecules due to its hydrophilic groups and may enhance intermolecular interactions within the protein matrix—such as ionic bonds, hydrogen bonds, hydrophobic interactions, and disulfide bonds—with meat proteins [9]. These interactions, facilitated by interpenetrating structures, contribute to the stabilization of the protein gel matrix and improve its WHC. Therefore, our study may provide insight into why the EM value was low, despite the reduced CL observed in PHP-added sausages.

### Sodium dodecyl sulfate–polyacrylamide gel electrophoresis

Figure 2 shows the SDS-PAGE results of sausages containing different concentrations of psyllium husk before and after cooking. Before cooking, no differences in the protein bands of the sausage according to PHP concentration were observed in the SDS-PAGE gel. In a previous study, the addition of psyllium husk addition to pork MP resulted in no evident bands in the SDS-PAGE gel [7], which is consistent with our results. This potentially arises from the primary component of PHP being carbohydrate, which may not directly affect the protein composition of MP or sausages. The myosin heavy chain (MHC) and actin bands were observed around 250 and 42 kDa, respectively. After cooking, a biopolymer band formed at the top of the SDS-PAGE gel as shown in the arrow in Figure 2, with the band intensity increasing with PHP addition. Conversely, as the PHP concentration increased, the intensity of the MHC and actin bands decreased. This reduction may be attributed to the involvement of MHC and actin in biopolymer formation. The aggregates at the top of the SDS-PAGE gel potentially formed through the participation of MHC, actin, and myosin light chain bands in disulfide bonds during heat treatment [7]. Therefore, the incorporation of PHP into the MP system may promote intramolecular interactions among MP proteins, allowing MHC and actin to participate in the formation of biopolymer bands through covalent linkages such as disulfide bonds, as suggested by previous studies [7,9]. These interactions may contribute to protein cross-linking and aggregation within the MP matrix.

### Texture profile analysis

Table 4 shows the textural properties of chicken sausages containing various levels of PHP. No differences in the textural characteristics of the sausages according to PHP concentration were noted (p>0.05). PHP, known for its excellent moisture retention capacity, can form a gel through moisture absorption. A previous study indicated that the consistency, firmness, and cohesiveness of the sols and gels of water-dispersed psyllium husk increased in a concentration-dependent manner according to the back-extrusion parameter results [25]. These findings demonstrated the gelation capacity of psyllium husk itself and suggested its potential application as a gelling agent in food systems. Previous studies have reported that the addition of psyllium husk to MP gel systems can enhance textural properties [7,10]. However, it is assumed that the following reason may explain why the addition of PHP had no impact on sausage texture. While the gel properties of PHP itself may be sufficient to enhance the textural properties of MP gels, they may be insufficient to improve the texture of meat products when incorporated into sausage formulations. In contrast to the general gel strength of MP, the gel firmness and hardness values of psyllium husk were reported to be lower than those observed in our sausage samples [25]. Therefore, in the sausage matrix, the gelation of PHP may improve water retention capacity, but unlike in the MP system, it may form a gel that is too weak to improve texture. A previous study reported no differences in the hardness, springiness, gumminess, chewiness, and cohesiveness of chicken sausages formulated with various dietary fibers (inulin, oats), including psyllium husk [24].

Other possible reasons for no difference in texture properties are as follows. The gelation properties of dietary fiber may be partially influenced by a negative correlation between water retention capacity and textural properties, which could be attributed to the high-water absorption capacity of dietary fibers or hydrocolloids. For instance, a negative correlation between hardness and both water-holding and water-retention capacities in heated psyllium husk gels, suggesting that gel structure is influenced by the hydration behavior of dietary fibers [25]. Similarly, the addition of hydrated oatmeal to pork sausages reduced textural properties while increasing WHC and reducing CL, indicating that WHC may affect texture, although the relationship appears to be complex and potentially controversial [28]. Overall, the reason why the addition of PHP did not enhance the textural properties of the sausages may be that the PHP gel itself was not strong enough to improve the sausage texture, and that the high-water retention capacity of PHP may not have induced a harder sausage texture. However, the addition of PHP does not appear to have interfered with protein-protein interactions, which can be supported by the results of SDS-PAGE (Figure 2) and the observation of the protein microstructure before digestion via LV-SEM (Figure 3).

### Protein digestibility during *in vitro* digestion

Table 5 presents the protein digestibility results of sausages containing different levels of PHP during *in vitro* digestion. Protein digestibility was evidently higher during the oral–gastrointestinal digestion stage than during the oral–gastric stage (p<0.05). Moreover, the protein digestibility of 1.0%–1.5%- PHP-added sausages exceeded that of 0.0%–0.5%-PHP-added sausages in the oral–gastric and oral–gastrointestinal digestion stages (p<0.05). To our knowledge, the effect of psyllium husk on *in vitro* protein digestibility in meat products has yet not been examined. In a previous study, rice pasta in which 50% of the formulation was replaced with psyllium husk gel showed reduced dry matter and carbohydrate digestibility compared with ordinary rice pasta; however, no difference in protein digestibility was observed [29]. In another study, *in vitro* protein digestibility was not affected even when 15% wheat bran was included in meat analogues manufactured using pea protein isolate or pea protein concentrate [30]. In contrast, another study discovered that the inclusion of resistant starch in pork muscle and salt-soluble protein gels resulted in higher protein digestibility during *in vitro* digestion [31]. In an *in vivo* study, feeding rats fiber-fortified milk containing a mixture of dietary fibers (including psyllium husk, wheat fiber, oat bran, and inulin) did not affect protein digestibility and reported that this result contradicts the general finding that dietary fiber reduces protein bioavailability [32]. Our *in vitro* digestion results revealed that protein digestibility increased with increasing PHP concentration compared with that of sausages without PHP (p<0.05). However, the protein digestibility may differ *in vivo*; however, this was not verified in our study. In conclusion, incorporating PHP into meat products does not appear to interfere with meat protein digestibility in an *in vitro* digestion system. However, future studies are necessary, as differences may exist between *in vitro* and *in vivo* results.

### Sodium dodecyl sulfate–polyacrylamide gel electrophoresis during *in vitro* digestion

Figure 4 displays the protein bands of sausages containing varying levels of PHP at different stages of *in vitro* digestion on SDS-PAGE gel. The “undigested” results shown in Figure 4A are consistent with the “after cooking” results depicted in Figure 2, as described above. As digestion progressed, the protein band was evidently digested during the oral–gastric digestion stage, with the protein bands>50 kDa gradually disappearing and the actin band faintly remaining. During oral–gastrointestinal digestion, the MHC and actin bands were completely digested, and protein bands with a molecular weight<37 kDa exclusively remained. A previous study reported that after *in vitro* digestion with pepsin and/or pancreatin, high-molecular-weight protein bands in pork and beef meat samples either decreased or disappeared, as high-molecular-weight proteins were degraded into smaller peptides or free amino acids [33]. Consequently, only low-molecular-weight fractions were observed in the previously reported results [33], which is consistent with the protein degradation pattern observed in our study. Most peptides with molecular weights<45 kDa, including actin (42 kDa), troponin T (35 kDa), and tropomyosin-α-chain (30 kDa), as well as peptides smaller than 15 kDa remained in the pepsin-digested pork proteins with or without resistant starch, suggesting that these bands exhibited greater resistance to pepsin [31]. Additionally, they also reported that the intensity of the actin band was lower in the resistant starch–porcine protein mixture gel, indicating increased protein hydrolysis [31]. In our study, the protein degradation pattern during the digestion process did not vary across different levels of PHP. Although *in vitro* protein digestibility increased with higher levels of PHP (p<0.05), no notable differences in protein degradation pattern were observed in the SDS-PAGE analysis. Therefore, the results of this study confirm that the addition of PHP did not adversely affect the protein digestibility of chicken sausages.

### Low-vacuum scanning electron microscopy during *in vitro* digestion

Figure 3 presents the surface microstructure of sausages containing different levels of PHP during *in vitro* digestion, as observed through LV-SEM. Before digestion, a denser and more uniform structure was observed with increasing PHP content, possibly because PHP acts as a filler between meat proteins, reducing water channels and minimizing both large and small cavities. In a previous study, the application of bamboo shoot dietary fiber to pork meat products resulted in a rough appearance and large void network in the microstructure of the product without the dietary fiber [23]. This phenomenon was related to greater water loss from the meat protein matrix. Dietary fiber addition increased the density of the protein network, reduced the voids in the water channels, and rendered the structure uniform and continuous. In this study, PHP addition also reduced CL and EM by making the meat protein network denser and more uniform, thereby reducing the pores in the water channels and preventing moisture from easily escaping. In addition, the improvement in the density of the three-dimensional surface microstructure of proteins may be attributed to the strengthening of cross-linking and covalent bonding between protein residues [34]. Our previous study also confirmed a tendency for disulfide bonding in MPs to increase with increasing amounts of psyllium husk [35].

As digestion progressed, the surface microstructure became rough, large and small cavities formed, and surface cracks increased. Although the surface structure of undigested sausage became denser with increasing PHP addition, it apparently suffered more damage during the digestion process due to simulated peristalsis and the action of digestive enzymes compared with PHP-free sausages. Even at the oral stage, where no proteolytic enzymes are present, the surface of sausages containing PHP—initially flat and compact before digestion—became rough and damaged, similar to sausages without PHP. This indicates that PHP may enhance subsequent water absorption in the meat protein matrix during *in vitro* digestion, leading to increased swelling and the collapse of the sausage structure. This might have increased structural accessibility to digestive enzymes, potentially improving digestibility during *in vitro* digestion. A previous study proposed that increasing *Abelmoschus manihot* gum concentration yielded smaller, more uniformly distributed protein particles after digestion, suggesting that the gum enhanced protein digestibility by increasing the effective contact area between meat proteins and digestive enzymes [36]. The gum absorbed additional water during *in vitro* digestion, leading to swelling and structural changes in meat proteins, thereby potentially enhancing their reactivity with digestive enzymes and improving digestibility [36].

### Spearman’s correlation coefficients

This study investigated the relationship between the physicochemical properties and *in vitro* protein digestibility of chicken sausages containing different levels of PHP, using Spearman’s correlation coefficients (Figure 5). The addition of PHP showed negative correlations with CL, lightness, EM, and springiness, whereas yellowness and protein digestibility exhibited positive correlations. These results suggest that PHP may contribute to improving the sausage’s WHC and protein digestibility. Protein digestibility showed negative correlations with both CL and EM, indicating that improving WHC by adding PHP may also influence protein digestibility. This supports the proposal by a previous study that additional water absorption by gums within a protein matrix during digestion can lead to structural expansion and alterations in protein structure, thereby enhancing protein digestibility [36]. Therefore, incorporating hydrocolloids, which absorb moisture and swell, into meat products may improve functional properties such as moisture retention during production. Moreover, during digestion, the additional water absorption and swelling may further enhance protein digestibility. Nevertheless, further analyses, such as proteomic studies, are required to elucidate the precise mechanisms of digestion.

## CONCLUSION

This study demonstrates that PHP influences the quality characteristics of chicken model sausages. As PHP concentration increased, the sausages exhibited decreased lightness and increased yellowness, along with reduced CL and EM, indicating an enhanced WHC. Importantly, the incorporation of 1.0%–1.5% PHP improved protein digestibility during *in vitro* digestion, while texture profile analysis revealed no alterations. These findings highlight PHP’s potential as a novel functional ingredient in meat products, offering benefits such as enhanced water retention and improved protein digestibility without any adverse effects on texture. Consequently, PHP presents a promising ingredient for developing healthier and more functional meat products. However, future studies on actual products will be necessary to confirm the practical application of PHP, including sensory evaluation.

## Figures and Tables

**Figure 1:**
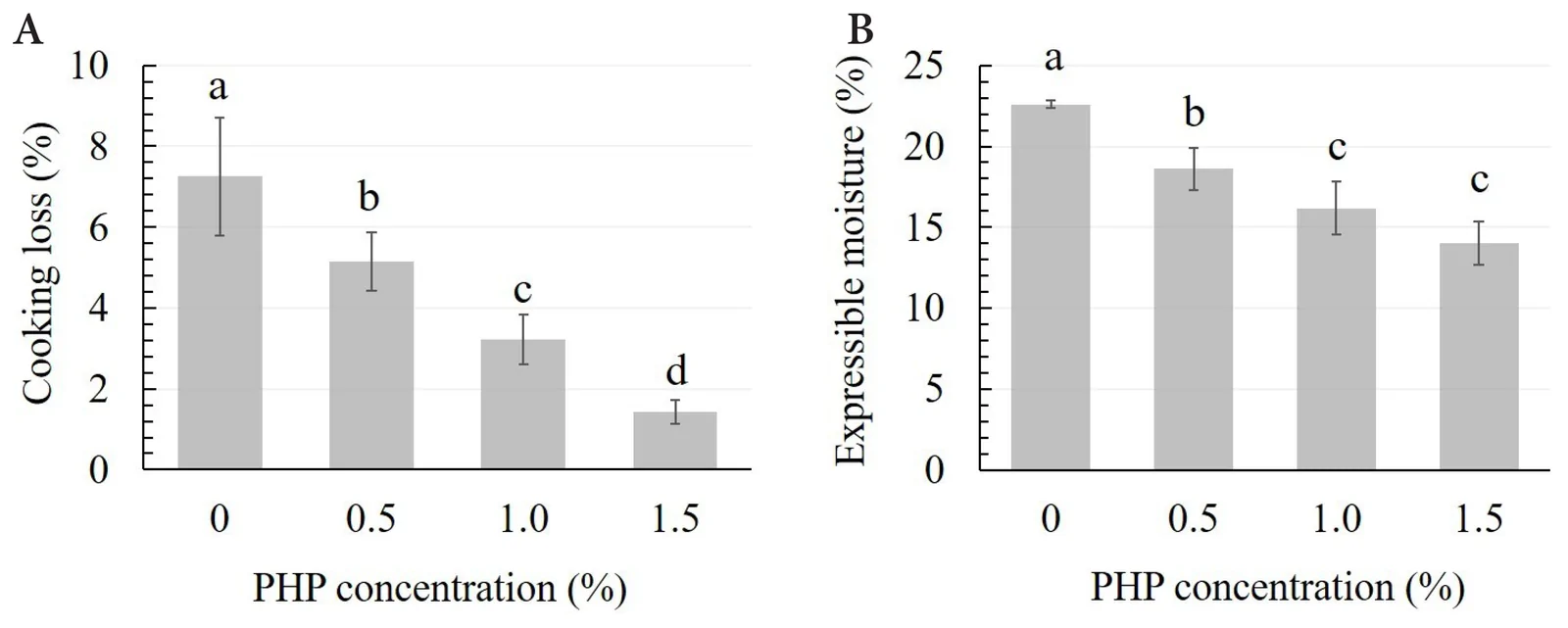
Cooking loss (A) and expressible moisture (B) of chicken model sausages containing different PHP concentrations (0%–1.5%). ^a–d^ Means with different superscripts in the same parameter are different (p<0.05). PHP, psyllium husk powder.

**Figure 2:**
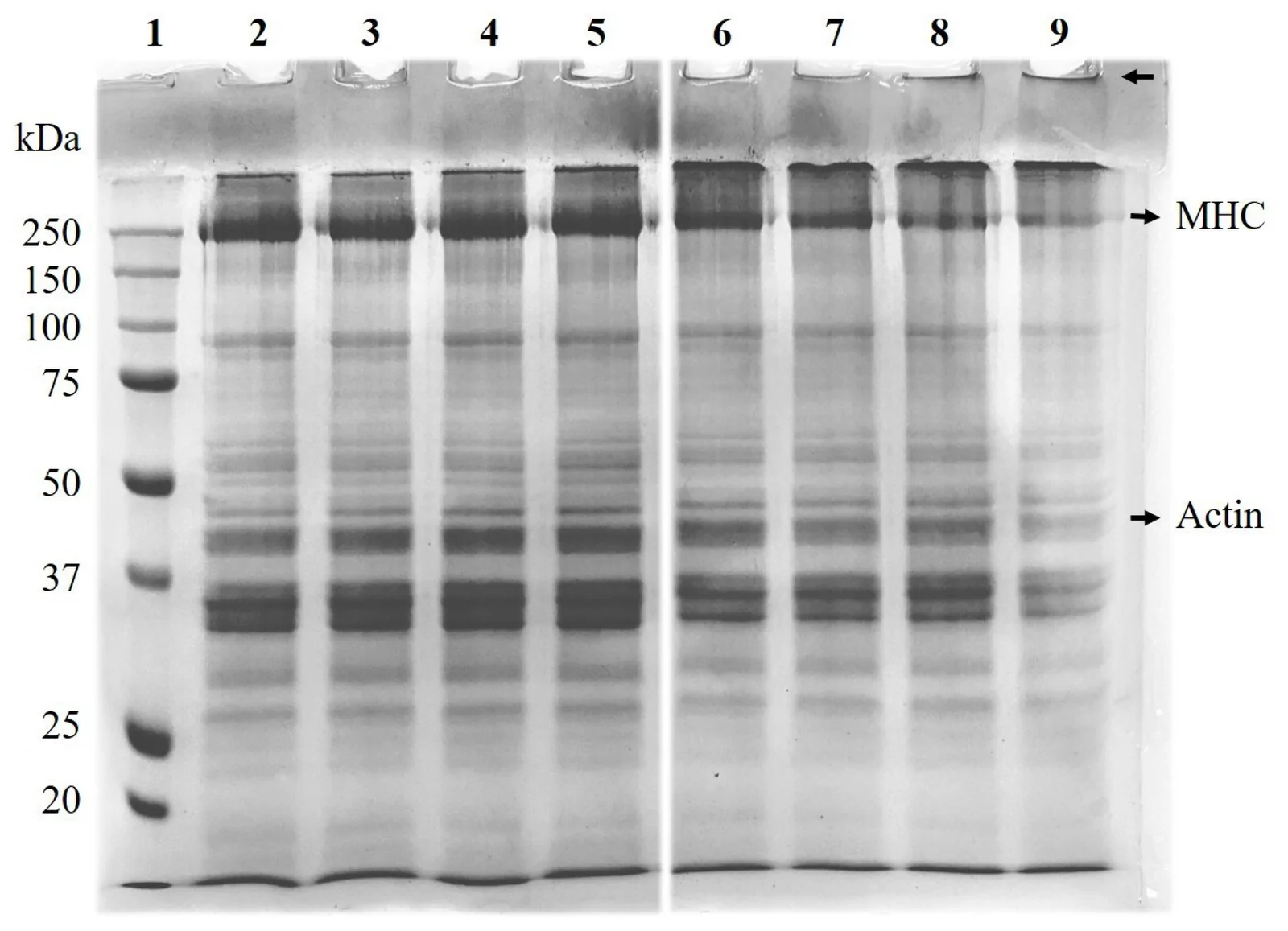
SDS-PAGE profile of chicken model sausages containing different PHP concentrations. SDS-PAGE was performed using 10% polyacrylamide separating gels and 4% stacking gel. Lane 1, protein molecular weight marker (250–20 kDa); Lanes 2–5, chicken sausage batter with different PHP concentrations (0%, 0.5%, 1.0%, and 1.5%); Lanes 6–9, cooked chicken sausages with different PHP concentrations (0%, 0.5%, 1.0%, and 1.5%). MHC, myosin heavy chain; SDS-PAGE, sodium dodecyl sulfate–polyacrylamide gel electrophoresis; PHP, psyllium husk powder.

**Figure 3:**
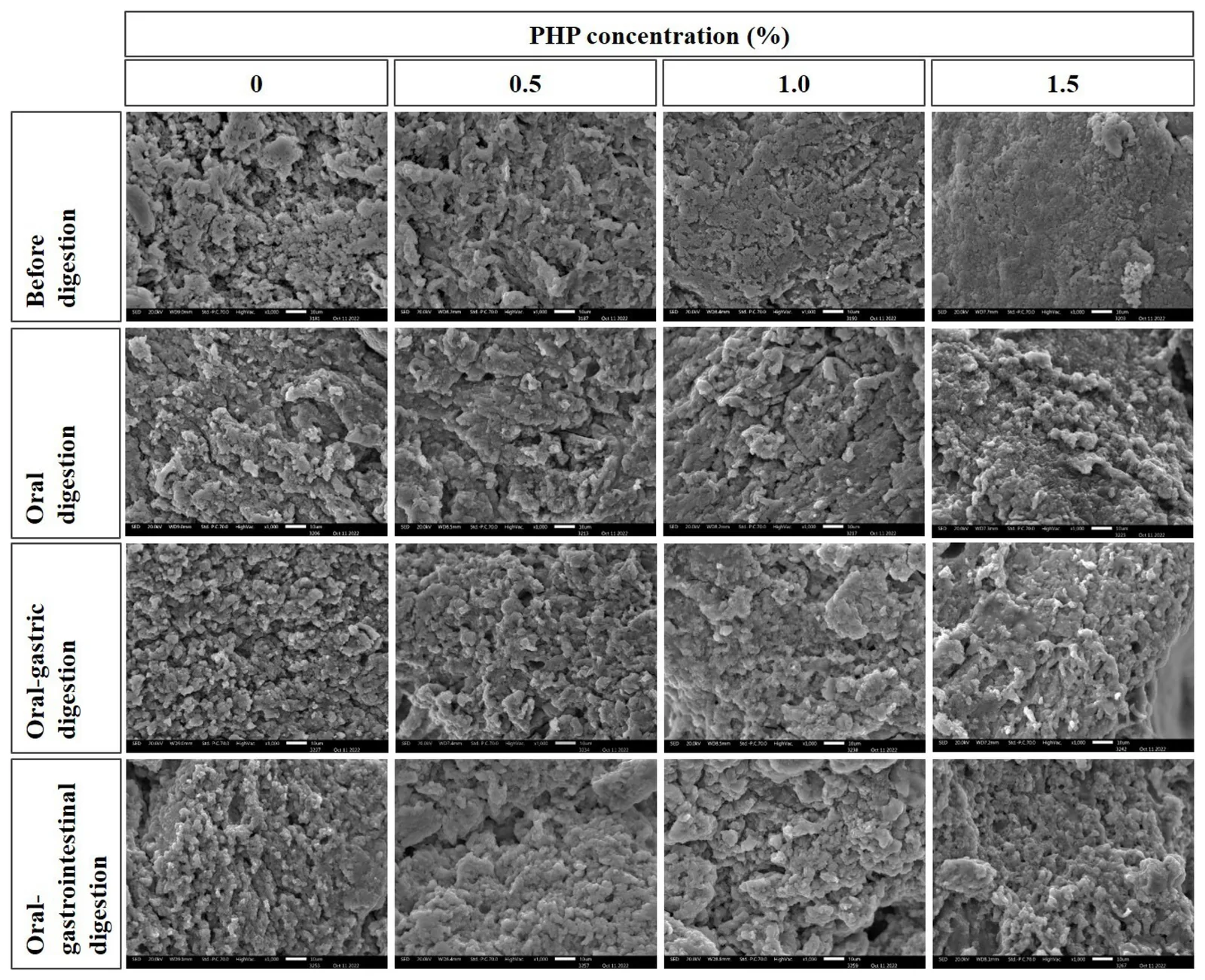
Microstructural images of chicken model sausages containing different PHP concentrations during *in vitro* digestion (×2,000 magnification). PHP, psyllium husk powder.

**Figure 4:**
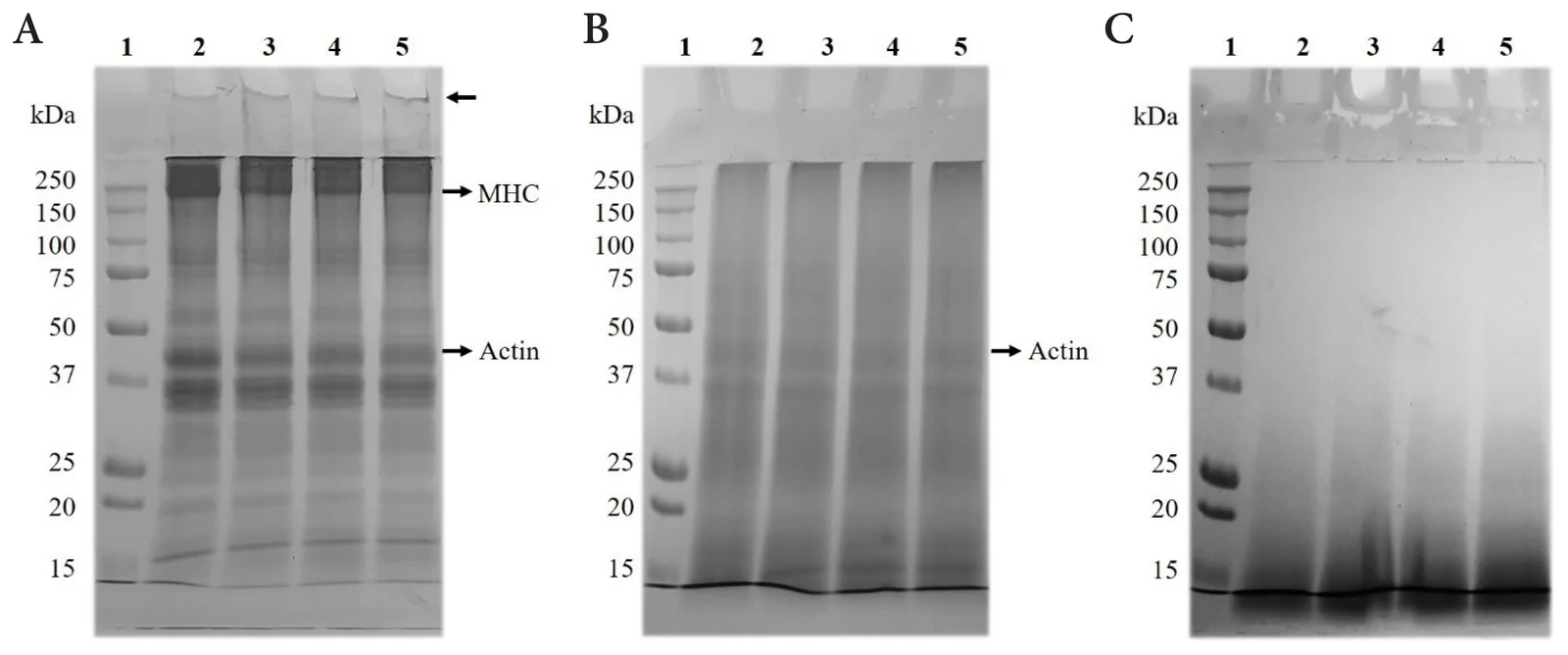
SDS-PAGE profile of chicken model sausages containing different PHP concentrations during *in vitro* digestion. (A) Before digestion; (B) After oral-gastric digestion; (C) After oral-gastrointestinal digestion. SDS-PAGE was performed using 12% polyacrylamide separating gels and 4% stacking gel. Lane 1, protein molecular weight marker (250–15 kDa); Lanes 2–5, cooked chicken sausages with different PHP concentrations (0%, 0.5%, 1.0%, and 1.5%). MHC, myosin heavy chain; SDS-PAGE, sodium dodecyl sulfate–polyacrylamide gel electrophoresis; PHP, psyllium husk powder.

**Figure 5:**
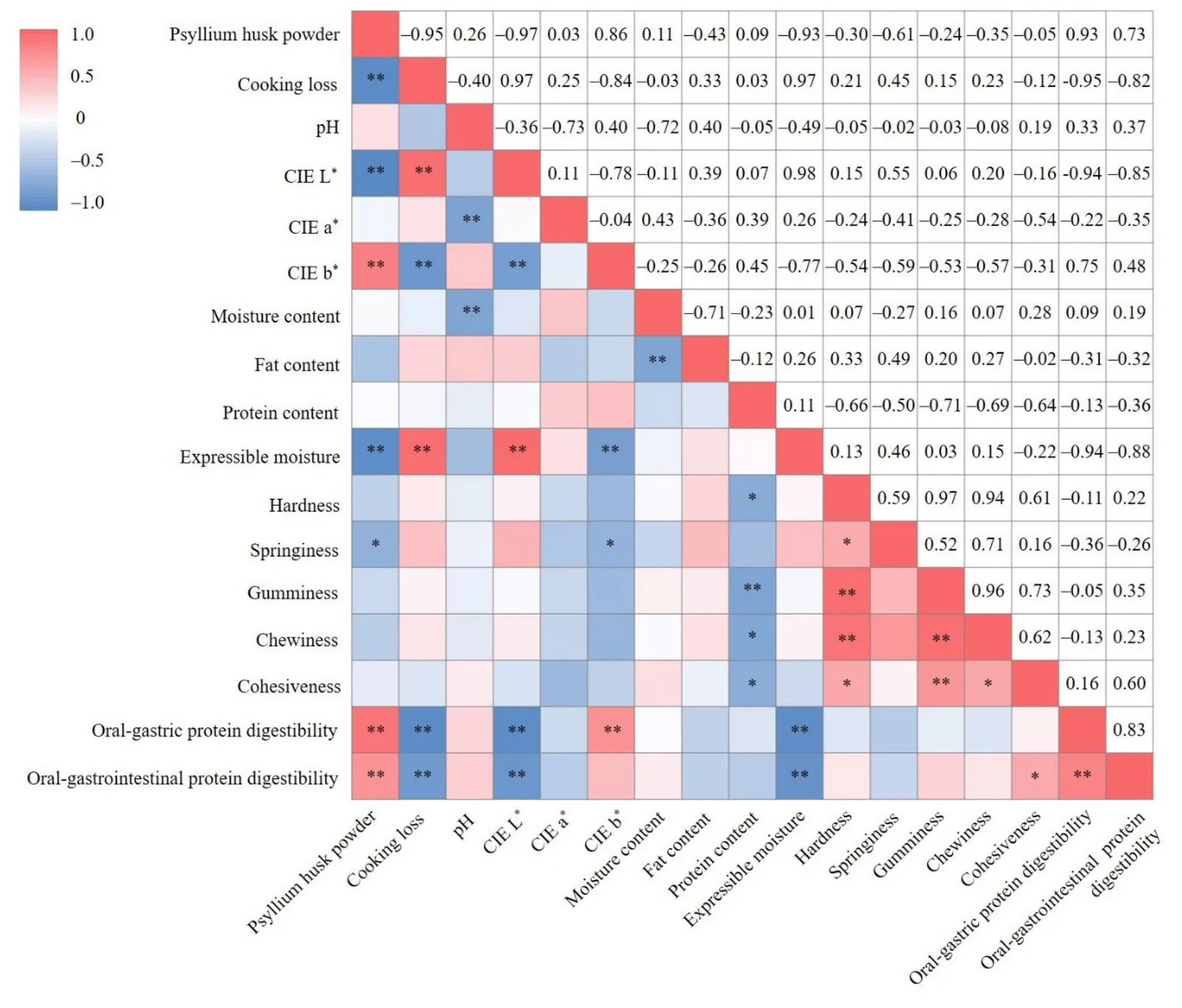
Heatmap of Spearman’s correlation coefficients between the addition of different PHP concentrations and the physicochemical properties and *in vitro* protein digestibility of chicken model sausages. The colors represent Spearman’s correlation coefficients, with red and blue indicating positive and negative correlations, respectively. Significant correlations are indicated as * p<0.05 and ** p<0.01. PHP, psyllium husk powder.

**Table 1:** Formulation of chicken model sausages containing different PHP concentrations

Ingredient (%)	PHP concentration (%)			

	0	0.5	1.0	1.5
Chicken breast	80.0	80.0	80.0	80.0
Ice water	18.2	18.2	18.2	18.2
Salt	1.50	1.50	1.50	1.50
Sodium tripolyphosphate	0.30	0.30	0.30	0.30
Sodium erythorbate	0.05	0.05	0.05	0.05
Sodium nitrite	0.015	0.015	0.015	0.015
PHP1)	0.00	0.50	1.00	1.50
Total	100.0	100.5	101.0	101.5

1) ≤300 μm.

PHP, psyllium husk powder.

**Table 2:** pH and color values of chicken model sausages containing different PHP concentrations

	PHP concentration (%)			

	0	0.5	1.0	1.5
pH	6.26±0.04^a^	6.28±0.05^a^	6.29±0.08^a^	6.28±0.08^a^
CIE L*	78.8±0.65^a^	76.7±0.43^b^	75.2±0.71^b^	73.2±1.18^c^
CIE a*	3.67±0.54^a^	3.73±0.52^a^	3.48±0.79^a^	3.75±0.54^a^
CIE b*	8.21±0.46^c^	8.64±0.39^bc^	9.28±0.53^ab^	9.59±0.23^a^

Data are the mean±standard deviation.

a–c Means with different superscripts in the same parameter are different (p<0.05).

PHP, psyllium husk powder.

**Table 3:** Proximate composition of chicken model sausages containing different PHP concentrations

	PHP concentration (%)			

	0	0.5	1.0	1.5
Moisture (%)	79.6±1.03^a^	79.7±1.63^a^	79.7±1.62^a^	79.7±1.65^a^
Protein (%)	17.6±0.45^a^	17.4±0.63^a^	17.9±1.01^a^	17.6±0.80^a^
Fat (%)	0.67±0.12^a^	0.48±0.21^a^	0.50±0.21^a^	0.46±0.22^a^

Data are the mean±standard deviation.

a Means with the same superscript in the same parameter didn’t differ (p>0.05).

PHP, psyllium husk powder.

**Table 4:** Texture profile analysis of chicken model sausages containing different PHP concentrations

	PHP concentration (%)			

	0	0.5	1.0	1.5
Hardness (gf)	3,465±267^a^	2,813±588^a^	2,723±577^a^	2,770±815^a^
Springiness (mm)	7.05±0.48^a^	6.64±0.77^a^	5.78±0.72^a^	5.92±0.97^a^
Gumminess	25.8±3.65^a^	21.3±7.38^a^	21.2±5.51^a^	21.4±8.06^a^
Chewiness	180±13.6^a^	140±51.5^a^	123±46.7^a^	132±72.1^a^
Cohesiveness	7.58±0.64^a^	7.35±1.17^a^	7.84±0.63^a^	7.55±0.53^a^

Data are the mean±standard deviation.

a Means with the same superscript in the same parameter didn’t differ (p>0.05).

PHP, psyllium husk powder.

**Table 5:** Protein digestibility of chicken model sausages containing different PHP concentrations

Protein digestibility (%)	PHP concentration (%)			

	0	0.5	1.0	1.5
Oral-gastric phase	15.6±0.18^B^^b^	16.2±0.18^B^^b^	17.1±0.31^B^^a^	17.7±0.63^B^^a^
Oral-gastrointestinal phase	45.8±0.49^A^^b^	46.8±1.39^A^^ab^	47.9±0.60^A^^a^	48.5±0.88^A^^a^

Data are the mean±standard deviation.

A,B Means with different superscripts in the same column are different (p<0.05).

a,b Means with different superscripts in the same row are different (p<0.05).

PHP, psyllium husk powder.

## Data Availability

Upon reasonable request, the datasets of this study can be available from the corresponding author.
